# Studies on Chemical Composition, Antimicrobial and Antioxidant Activities of Five *Thymus vulgaris* L. Essential Oils

**DOI:** 10.3390/molecules200712016

**Published:** 2015-07-01

**Authors:** Emilia Mancini, Federica Senatore, Donato Del Monte, Laura De Martino, Daniela Grulova, Mariarosa Scognamiglio, Mejdi Snoussi, Vincenzo De Feo

**Affiliations:** 1 Department of Pharmacy, University of Salerno, Via Giovanni Paolo II, 132, Fisciano 84084, Salerno, Italy; E-Mails: emancini@unisa.it (E.M.); federica.senatore90@gmail.com (F.S.); blackwhites@interfree.it (D.D.M.); ldemartino@unisa.it (L.D.M.); 2 Department of Ecology, Faculty of Humanities and Natural Sciences, University of Presov, 17 November street, Presov 08116, Slovak; E-Mail: dgrulova@gmail.com; 3 Department of Industrial Engineering, University of Salerno, 1 Via Giovanni Paolo II, 132, Fisciano 84084, Salerno, Italy; E-Mail: mscognamiglio@unisa.it; 4 Laboratory of Water Treatment and Reuse, Water Research and Technologies Center, Technopark of Borj-Cedria, BP 273, Soliman 8020, Tunisia; E-Mail: snmejdi@yahoo.fr

**Keywords:** *Thymus vulgaris*, essential oil, antimicrobial activity, antioxidant activity, phenolic compounds

## Abstract

This study is aimed at assessing the essential oil composition, total phenolic content, antimicrobial and antioxidant activities of *Thymus vulgaris* collected in five different area of the Campania Region, Southern Italy. The chemical composition of the essential oils was studied by GC-flame ionization detector (FID) and GC/MS; the biological activities were evaluated through determination of MIC and minimum bactericidal concentration (MBC) and evaluation of antioxidant activity. In total, 134 compounds were identified. The oils were mainly composed of phenolic compounds, and all oils belonged to the chemotype thymol. The antimicrobial activity of the five oils was assayed against ten bacterial strains. The oils showed different inhibitory activity against some Gram-positive pathogens. The total phenol content in the essential oils ranged from 77.6–165.1 mg gallic acid equivalents (GAE)/g. The results reported here may help to shed light on the complex chemotaxonomy of the genus *Thymus*. These oils could be used in many fields as natural preservatives of food and as nutraceuticals.

## 1. Introduction

In the last few years, there has been a strong interest in natural products obtained by plants as drugs, pharmaceuticals, perfumery products, cosmetics and food additives. Among these products, the essential oils from aromatic plants have received more attention for their different biological activities [1]. The compositions of the essential oils are very much influenced by intrinsic factors, such as species, cultivar, clone and ecotype, and ecological factors, such as geographical origin, climatic conditions, soil, biotic and technological factors, cultivation techniques, types of collection processes, storage conditions of raw materials and processing technologies [2]. For this reason, wild and cultivated plants of the same species, but from different contexts can express different features and chemical compositions. In this article, attention was focused on *Thymus vulgaris* L. (Lamiaceae). The genus *Thymus* comprises about 300 species of perennial aromatic, herbaceous plants with many subspecies, varieties, subvarieties and forms. *T. vulgaris* is the most widespread species of thyme in Italy and is a pleasant smelling perennial shrub that is present in the Mediterranean area with at least six different chemotypes [2]. The *T. vulgaris* essential oils have been found to display different biological properties [3]. Some papers are dedicated to the antimicrobial activity of the essential oil of *T. vulgaris* and of its single constituents. Moreover, the antioxidant property of thyme make its helpful for food safety [4,5,6].

This study is aimed at assessing the essential oil composition, total phenolic content, antimicrobial and antioxidant activities of *Thymus vulgaris* collected in five different area of the Campania Region, Southern Italy.

## 2. Results and Discussion

### 2.1. Essential Oil Yield and Composition

Hydrodistillation of the aerial parts of five samples of *T. vulgaris* harvested in five distinct areas in Campania, *i.e.*, the campus of the University of Salerno (S), Frigento (F), Contrada La Francesca (LF), Morigerati (M) and Zungoli (Z), gave yellow essential oils characterized by a typical odor, with yields of 0.068, 0.070, 0.092, 0.019 and 0.081% (*v*/*w*, on a fresh weight basis) for the samples from S, F, LF, M and Z, respectively. Table 1 shows the chemical composition of the five essential oils; the compounds are listed according to their elution order on an HP-5 MS capillary column. Altogether, 134 compounds were identified, 47 for *T. vulgaris* from S, 82 for F, 78 for LF, 70 for M and 44 for Z, accounting for 84.5%, 82.7%, 86.5%, 79.7% and 73.6% of the total oil compositions, respectively. The phenolic compounds highly predominated in all essential oils. In all oils, thymol (46.2%–67.5%), carvacrol (5.7%–7.3%) and caryophyllene oxide (1.7%–7.3%) were the most abundant compounds.

**Table 1:** Chemical composition of the essential oils isolated from the aerial part of *Thymus vulgaris* collected at the campus of the University of Salerno (S), Frigento (F), Contrada la Francesca (LF), Morigerati (M) and Zungoli (Z).

No.	Compound	Ri ^a^	Ri ^b^	S%	F%	LF%	M%	Z%	Identification
1	β-Pinene	987	1118	- ^c^	t ^d^	-	-	-	1,2,3
2	δ-3-Carene	1011	1159	0.1	-	t	0.1	-	1,2,3
3	α-Terpinene	1011	1188	-	0.1	-	-	t	1,2,3
4	*m*-Cymene	1019	1280	t	t	t	t	t	1,2,3
5	β-Phellandrene	1023	1218	-	-	t	t	-	1,2,3
6	1,8-Cineole	1027	1213	-	t	t	t	-	1,2,3
7	γ-Terpinene	1055	1255	t	t	t	t	-	1,2,3
8	*cis-*Sabinene hydrate	1066	1556	0.1	0.2	0.5	0.2	-	1,2
9	*p*-Cymene	1086		-	t	t	-	-	1,2,3
10	*trans*-Sabinene hydrate	1094	1474	-	-	t	-	-	1,2
11	Linalool	1097	1553	0.5	1.8	2.7	2.3	0.3	1,2,3
12	Myrcenol	1111		-	-	0.2	-	-	1,2
13	Dehydro-sabina ketone	1119		-	t	-	-	-	1,2
14	*iso*-3-Thujanol	1137		-	0.1	t	-	-	1,2
15	3-Thujanol	1163		0.1	-	-	-	-	1,2
16	Borneol	1164	1719	-	0.5	0.2	0.5	0.3	1,2,3
17	Terpinen-4-ol	1174	1611	t	0.4	0.4	0.7	0.3	1,2,3
18	*neo*-*iso*-Dihydrocarveol	1189		-	0.2	0.3	0.1	-	1,2
19	α-Terpineol	1190	1706	-	-	-	0.1	-	1,2,3
20	*p*-Cymen-4-ol	1199		-	-	t	-	-	1,2
21	*cis*-4-Caranone	1200		-	t	-	-	-	1,2
22	γ-Terpineol	1202		0.1	0.3	-	0.2	0.2	1,2,3
23	*trans*-Carveol	1209	1845	-	t	-	-	-	1,2
24	Coahuilensol methyl ether	1214		t	t	t	0.1	0.1	1,2
25	*cis*-Sabinene hydrate acetate	1217		-	t	-	-	-	1,2
26	*cis*-Carveol	1224		-	-	t	-	-	1,2
27	2-prenyl-Cyclopentanone	1226		-	-	t	-	-	1,2
28	*cis*-*p*-Mentha-1(7)8-dien-2-ol	1227		-	t	-	-	-	1,2
29	*cis*-Pulegol	1231		-	-	t	-	-	1,2
30	(E)-Ocimenone	1233		-	t	-	-	-	1,2
31	*trans*-Crysanthenyl acetate	1238		-	-	t	-	-	1,2
32	Chavicol	1247		-	-	t	-	-	1,2
33	Carvacrol methyl ether	1247		-	t	-	-	-	1,2
34	Geraniol	1253	1857	0.2	0.7	0.2	0.6	0.1	1,2,3
35	*trans*-Myrtanol	1268		-	2.3	-	-	1.7	1,2
36	Oxygenated monoterpene	1268		0.2	-	0.1	-	-	
37	*cis*-Crysanthenyl acetate	1270		-	-	0.2	-	-	1,2
38	Citronellyl formate	1276		2.5	-	-	1.9	-	1,2
39	Ethyl-2-octynoate	1283		-	-	1.8	-	-	1,2
40	*cis*-Verbenyl acetate	1283		-	0.1	-	-	0.1	1,2
41	*p*-Cymen-7-ol	1285	2067	-	-	-	0.2	-	1,2
42	Thymol	1291	2198	63.0	52.4	67.5	50.2	46.2	1,2,3
43	Carvacrol	1311	2239	6.1	7.1	5.7	7.3	6.5	1,2,3
44	Oxygenatedmonoterpene	1311		-	-	-	-	0.2	
45	(*Z*)-Patchenol	1317		0.2	0.2	0.1	0.3	t	1,2
46	Oxygenated monoterpene	1317		-	0.1	-	-	-	
47	Phenolic derivate	1322		-	-	t	-	-	
48	(*E*)-Patchenol	1325		-	-	-	t	-	1,2
49	Piperitenone	1325	1949	-	0.1	t	t	-	1,2
50	Oxygenated monoterpene	1327		0.1	-	-	-	-	
51	Carvacrol acetate	1354		-	0.1	t	-	-	1,2
52	Thymol acetate	1354		0.1	-	0.1	t	0.1	1,2
53	Eugenol	1358	2186	t	t	t	t	t	1,2,3
54	Piperitenone oxide	1366	1983	0.1	t	0.1	t	1.4	1,2
55	Linalool isobutanoate	1375		-	-	-	0.2	0.1	1,2
56	Isobornyl propanoate	1376		0.1	0.2	0.1	-	-	1,2
57	*trans*-Myrtanol acetate	1379		-	-	t	-	-	1,2
58	Geranyl acetate	1382		-	0.1	-	-	-	1,2,3
59	Methyl eugenol	1403		-	t	-	-	-	1,2,3
60	*trans*-α-Ambrinol	1415		-	t	-	-	-	1,2
61	(*E*)-Caryophyllene	1419	1612	0.2	0.6	0.8	1.1	-	1,2,3
62	β-Copaene	1427		-	t	t	t	-	1,2
63	(*E*)-α-lonone	1431		-	-	t	-	-	1,2
64	Amorpha-4,11-diene	1453		-	-	0.2	-	-	1,2
65	α-Terpinyl isobutanoate	1471		-	-	1.1	-	-	1,2
66	Geranyl propanoate	1473		2.2	1.0	-	0.5	1.6	1,2
67	γ-Gurjunene	1476		-	0.1	t	0.2	-	1,2
68	γ-Muurolene	1480	1704	-	t	t	t	-	1,2
69	(*E*)-β-Ionone	1487	1957	0.1	0.1	0.1	0.1	0.2	1,2
70	*cis*-β-Guaiene	1491		-	t	-	t	-	1,2
71	*tran*s-Muurola-4(14),5-diene	1492		t	-	t	-	-	1,2
72	*cis*-Cadina-1,4-diene	1495		-	-	-	-	0.1	1,2
73	*epi*-Cubebol	1495	1900	0.1	0.1	0.1	0.1	-	1,2
74	γ-Amorphene	1497		-	t	-	t	0.1	1,2
75	α-Muurolene	1500	1740	0.1	0.1	0.1	-	-	1,2
76	β-Himalachene	1501		-	-	-	0.1	-	1,2
77	Lavandulyl isovalerate	1507		-	0.1	-	0.1	-	1,2
78	Oxygenated sesquiterpene	1510		0.6	0.1	0.1	0.3	0.5	
79	γ-Cadinene	1514	1766	0.2	0.3	0.1	0.3	-	1,2
80	Cubebol	1517	1957	-	0.1	0.1	0.1	0.1	1,2
81	Laciniata furanone G	1519		-	-	t	-	-	1,2
82	*trans*-Calamenene	1522		-	0.2	-	t	-	1,2
83	δ-Cadinene	1524	1773	0.4	0.4	0.4	0.6	0.1	1,2
84	Zonarene	1526	1729	-	-	-	0.1	-	1,2
85	*trans*-Cadina*-*1,4-diene	1532		-	-	0.1	t	-	1,2
86	γ-Cuprene	1532		-	t	-	-	-	1,2
87	α-Cadinene	1537	1743	-	0.1	t	0.1	-	1,2
88	α-Calacorene	1542	1941	t	t	t	0.1	-	1,2
89	α-Agarofuran	1550	1916	-	-	-	t	-	1,2
90	(*E*)-Nerolidol	1559	2050	0.6	0.5	0.3	-	0.7	1,2
91	β-Calacorene	1563		t	t	t	t	-	1,2
92	Caryophyllene derivative	1566		-	-	t	-	-	
93	Germacrene-d-4-ol	1572		0.1	-	t	-	-	1,2
94	Spathulenol	1578	2144	0.1	0.2	0.1	0.2	0.3	1,2,3
95	Caryophyllene oxide	1584	2008	2.2	6.5	1.7	7.3	7.1	1,2,3
96	β-Copaen-4-α-ol	1589		-	-	t	-	-	1,2
97	*allo*-Cedrol	1591		-	t	-	-	-	1,2
98	(*E*)-Dihydro-apofarnesol	1595		-	-	-	t	-	1,2
99	*n*-Hexadecane	1600		0.1	0.1	t	-	t	1,2
100	Geranyl 2-methyl butanoate	1600		0.1	0.1	t	0.2	0.1	1,2
101	β-Atlantol	1608		-	-	-	t	-	1,2
102	Humulene epoxide II	1610	2071	t	0.1	-	-	0.1	1,2
104	1,10-di-*epi*-Cubenol	1616		t	-	-	-	-	1,2
104	α-Corocalene	1623		-	t	t	t	-	1,2
105	10-*epi*-γ-Eudesmol	1625	2127	0.2	0.1	-	t	-	1,2
106	1-*epi*-Cubenol	1628	2088	0.1	0.1	-	0.1	-	1,2
107	(*E*)-Sesquilavandulol	1631		0.5	0.4	0.2	0.1	0.5	1,2
108	Selina-1,3,7-(11)-trien-8-one	1633		-	-	-	0.2	-	1,2
109	Oxygenated sesquiterpene	1634		0.1	-	-	-	-	
110	Caryophylla-4(12),8(13)-dien-5α-ol	1637		0.1	0.5	0.1	0.5	0.5	1,2
111	Hinesol	1641	2210	-	-	0.2	t	-	1,2
112	*epi*-α-Cadinol	1642		0.6	0.6	-	0.5	0.8	1,2
113	α-Muurolol	1645		-	-	0.1	-	-	1,2
114	Agarospirol	1647		0.4	0.3	-	-	-	1,2
115	Cedr-8(15)-en-9-α*-*ol	1649		-	-	-	0.3	-	1,2
116	*cis*-Guaia-3,9-dien-11-ol	1651	2269	-	0.1	-	-	-	1,2
117	α-Cadinol	1656	2255	0.4	0.5	0.1	0.4	0.7	1,2
118	14-hydroxy-(*Z*)-Caryophyllene	1658	2357	-	-	-	1.6	0.3	1,2
119	*trans*-Calamenen-10-ol	1668		-	-	t	-	-	1,2
120	14-hydroxy-9-*epi*-(*E*)-Caryophyllene	1672		0.5	1.0	0.2	-	1.3	1,2
121	Cadalene	1676	2256	t	0.1	t	0.3	0.1	1,2
122	Germacra-4(15),5,10(14)-trien-1-α-ol	1680		-	0.1	0.1	0.3	-	1,2
123	Kushinol	1681		-	-	-	0.1	-	1,2
124	Eudesma-4(15),7-dien-1-β-ol	1686	2391	0.2	0.3	t	0.3	0.4	1,2
125	*cis*-14-*nor*-Muurol-5-en-4-one	1689		-	-	-	-	0.1	1,2
126	(2*Z*,6*Z*)-Farnesol	1698		0.4	0.3	-	-	0.2	1,2
127	*n*-Heptadecane	1700		0.1	0.2	t	-	-	1,2
128	*cis*-Thujopsene	1707		0.1	-	-	-	-	1,2
129	14-hydroxy-α-Humulene	1708		-	0.1	-	-	-	1,2
130	Oplopanone	1738	2568	t	t	t	t	t	1,2
131	1-Octadecene	1751		t	-	-	-	-	1,2
132	2-α-hydroxy-Amorpha-4,7(11)-diene	1757		-	-	-	t	-	1,2
133	*n*-Octadecane	1800		0.1	0.1	t	t	t	1,2
134	6,10,14-trimethyl-2-Pentadecanone	1844		0.1	0.1	-	-	0.1	1,2
	**Total**			**84.5**	**82.7**	**86.5**	**79.7**	**73.6**	
	**Monoterpenes**			**0.1**	**0.1**	**-**	**0.1**	**-**	
	**Oxygenated monoterpenes**			**6.2**	**8.3**	**6.1**	**7.8**	**6.3**	
	**Sesquiterpenes**			**0.9**	**1.9**	**1.7**	**2.9**	**0.4**	
	**Oxygenated sesquiterpenes**			**7.4**	**12.2**	**3.4**	**2.9**	**14.1**	
	**Phenolic compounds**			**75.5**	**66.7**	**77.4**	**65.8**	**68.8**	

^a^ Ri^a^ and Ri^b^ are the Kovats retention indices determined relative to a series of *n-*alkanes (C_10_–C_35_) on the apolar HP-5 MS and the polar HP Innowax capillary columns, respectively; ^b^ identification method: 1: comparison of the Kovats retention indices with published data; 2: comparison of mass spectra with those listed in the NIST 02 and Wiley 275 libraries and with published data; 3: coinjection with authentic compounds; ^c^ -: not detected; ^d^ trace (<0.1%).

### 2.2. Minimum Inhibitory Concentrations

The minimum inhibitory concentration (MIC) and the minimum bactericidal concentration (MBC) values of the five essential oils against ten selected microorganisms are reported in Table 2. The five essential oils showed different inhibitory activity against the Gram-positive pathogens. Among the Gram-negative bacteria, *E. coli* was affected by the oil of Frigento (F). *Staphylococcus epidermidis* was the more sensitive bacterial strain.

**Table 2:** MIC and MBC values (μg/mL) of the five essential oils of *Thymus vulgaris* obtained from samples collected at the campus of University of Salerno (S), Frigento (F), Contrada la Francesca (LF), Morigerati (M) Zungoli (Z) and MIC of the reference compound, chloramphenicol. Results are the mean of three experiments.

	S		F		LF		M		Z		C
**Bacterial Strain**	**MIC**	**MBC**	**MIC**	**MBC**	**MIC**	**MBC**	**MIC**	**MBC**	**MIC**	**MBC**	
*Bacillus cereus* ATCC 177	25	50	25	-	50	100	50	-	50	100	12.5
*Bacillus subtilis* ATCC 633	100	-	12.5	25	50	100	25	50	50	-	12.5
*Staphylococcus aureus* ATCC 25923	>100	-	50	100	100	>100	100	>100	100	-	25
*Staphylococcus epidermidis* ATCC 12228	25	50	6.25	12.5	50	-	25	-	12.5	>25	3.12
*Streptococcus faecalis* ATCC 29212	50	100	25	50	100	-	100	-	100	>100	25
*Escherichia coli* ATCC 25922	50	100	25	50	50	100	50	-	50	100	12.5
*Klebsiella pneumoniae* ATCC 10031	>100	-	50	100	100	-	100	-	100	>100	50
*Proteus vulgaris* ATCC 13315	100	-	50	-	100	-	50	100	50	100	25
*Pseudomonas aeruginosa* ATCC 27853	100	>100	100	-	>100	-	100	>100	100	>100	100
*Salmonella typhi* Ty2 ATCC 19430	100	>100	50	100	100	-	100	-	100	>100	6.25

MIC: minimal inhibitory concentration (μg/mL); MBC: minimal bactericidal concentration (μg/mL); C: chloramphenicol.

### 2.3. Total Phenolic Content

**Figure 1:**
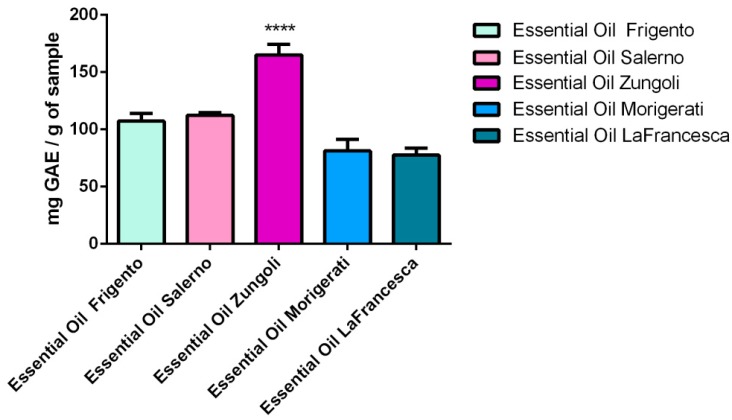
Total phenolics content of five essential oils from *Thymus vulgaris*. Data are expressed as mg of gallic acid equivalents (GAE)/g of essential oil. Each value in the table was obtained by calculating the mean of three experiments ± SD; Dunnett’s test: **** *p* < 0.0001 *vs*. all oils.

The concentration of total phenols was determined in the five essential oils of *T. vulgaris* plants. In Figure 1, the results of the colorimetric analysis are given; they were derived from the absorbance values of the oil solutions compared to the standard solutions of gallic acid equivalents (standard curve equation: y = 0.00119x − 0.00532, *r*^2^ = 0.9996). The total phenol content of the five oils ranged from 77.6–165.1 mg gallic acid equivalents (GAE)/g of sample (essential oil). The essential oil from Zungoli contained significantly higher total phenols (165.1 mg GAE/g) than the other oils*.*

### 2.4. Free Radical-Scavenging Capacity

The antioxidant activity of *T. vulgaris* essential oils was assessed by DPPH assay, evaluating the H-donating or radical scavenging ability of the oils using the stable radical 2,2-diphenyl-1-picrylhydrazyl (DPPH) as a reagent. Table 3 shows the concentrations that led to 50% inhibition (IC_50_) for three of the studied thyme oils (data for essential oils from Zungoli and Morigerati are unavailable). Ascorbic acid was used as a standard antioxidant. In this study, the IC_50_ values of the studied oils were less than the value of the reference antioxidant ascorbic acid (IC_50_ values of 3.10 ± 1.13 μg/mL) [7].

**Table 3:** IC_50_ value of three essential oils of *Thymus vulgaris* and ascorbic acid, after 60 min.

Sample/Essential Oil	IC_50_ Value (μg/mL)
Ascorbic acid (5 μg/mL)	3.10 ± 1.132
Essential Oil Frigento	64.93 ± 1.30
Essential oil Campus of the University of Salerno	28.95 ± 1.11
Essential oil Contrada La Francesca	58.25 ± 1.14

Data are the mean ± SD of five experiments.

The essential oil composition of the five *T. vulgaris* populations appeared similar, and the oils belonged to the same chemotype. Indeed, the five oils were characterized by high percentages of phenols and can be classified as oils belonging to the thymol chemotype. The variations between the main compounds of thyme essential oil can be explained by the biosynthetic relationship between the two phenols.

The metabolic pathway for the carvacrol and thymol formation begins with the autoxidation of γ-terpinene to *p*-cymene and the subsequent hydroxylation to thymol [8]. In the literature, it was reported that *Thymus vulgaris* has a chemical polymorphism with six different chemotypes that show spatial segregation in nature: phenolic chemotypes (thymol and carvacrol) and non-phenolic chemotypes (geraniol, α-terpineol, linalool and *trans*-thujan-4-ol/terpinen-4-ol) [9].

The different antimicrobial activity of these oils might be due to the little variation in their chemical profile. In the literature, it was reported that various chemical compounds have direct activity against many species of bacteria, such as terpenes and a variety of aliphatic hydrocarbons (alcohols, aldehydes and ketones). The lipophilic character of their hydrocarbon skeleton and the hydrophilic character of their functional groups are of main importance in the antimicrobial action of essential oils components, and the importance of the hydroxyl group of phenolic structures has been confirmed.

Moreover, the aldehyde group conjugated to a carbon-to-carbon double bond is a highly electronegative arrangement, which may explain their activity, suggesting a proportional increase of the antibacterial activity with electronegativity. The activity increased with the length of the carbon chain. Secondly, there is some evidence that minor components have a critical part to play in antibacterial activity, possibly by producing a synergistic effect between other components. This has been found for sage, some species of *Thymus* and oregano [10]. The appreciable total phenol contents of the five essential oils can also contribute to the antimicrobial activity. Ahmad and coworkers [3] reported that synergistic and additive interactions occur between the major and minor constituents present in the essential oil of *Thymus vulgaris*, and in this way, the antimicrobial efficacy of the essential oil could be enhanced.

Our data concerning total phenolic content are in line with previous research [11,12], which reports that the phenolic compounds are the main compounds in the thyme essential oil. The variation of the total phenolic content may be due to environmental conditions, such as soil composition and nitrogen content, which can modify the constituents of the plant [13,14].

The moderate antioxidant activity of the essential oil from the campus of the University of Salerno is probably due to the high amount of oxygenated compounds (phenolic compounds, 75.5%; oxygenated monoterpenes, 6.4%; oxygenated sesquiterpenes, 7.4%) and to the total phenolic content (112.3 mg GAE/g of sample). Our results are in agreement with previous studies, which showed that greater antioxidant potential of several *Thymus* species’ essential oils could be related to the nature of the phenolic compounds and their hydrogen ability. Besides, such activity could be ascribable to the oxygenated compounds, such as carvacrol and thymol. Moreover, the activities of essential oils of *Thymus* species depend on several structural features of the molecules and are primarily attributed to the high reactivity of the hydroxyl group substituent [15]. Moreover, the essential oils that contain oxygenated monoterpenes and/or sesquiterpenes have been reported for their greater antioxidative properties [1].

## 3. Experimental Section

### 3.1. Plant Material

*Thymus vulgaris* samples were collected in five localities in Campania, Southern Italy: the campus of the University of Salerno (S), Frigento (F), Contrada La Francesca (LF), Morigerati (M) and Zungoli (Z). Representative homogeneous samples of each population were collected during the balsamic time, corresponding to the flowering stage. The plants were identified by Vincenzo De Feo, and voucher specimens (DFE222/2013, DFE 218/2013, DFE 219/2013 and DFE234/2013 for S, F, LF, M and Z, respectively) have been deposited in the Herbarium of the Medical Botany Chair of the University of Salerno.

### 3.2. Isolation of the Volatile Oils

One hundred grams of fresh aerial parts of each sample were ground in a Waring blender and then subjected to hydrodistillation for 3 h according to the standard procedure described in the European Pharmacopoeia [16]. The oils were solubilized in *n*-hexane, dried over anhydrous sodium sulfate and stored under N_2_ at +4 °C in the dark until tested and analyzed. The calculated essential oil yield was expressed in % (*v/w*), based on the weight of the fresh plant material. All extractions were done in triplicate.

### 3.3. GC-FID Analysis

The gas chromatography-flame ionization detector (GC-FID) analysis was carried out on a Perkin-Elmer Sigma-115 gas chromatograph equipped with a flame ionization detector (FID) and a data handling processor. The separation was achieved using an apolar HP-5 MS fused-silica capillary column (30 m × 0.25 mm i.d., 0.25-μm film thickness); column temperature: 40 °C, with 5 min initial hold, then to 270 °C at 2 °C/min and, finally, at 270 °C for 20 min; injection mode: splitless (1 μL of a 1:1000 *n*-pentane solution). Injector and detector temperatures were 250 °C and 290 °C, respectively. Analysis was also run by using a fused silica HP Innowax polyethylene glycol capillary column (50 m × 0.20 mm i.d., 0.25-μm film thickness). In both cases, helium was used as the carrier gas (1.0 mL/min). The relative essential oil contents of the components were obtained by peak area normalization, without calculating response factors.

### 3.4. GC/MS Analysis

The gas chromatography-mass spectroscopy (GC/MS) analysis was performed with an Agilent 6850 Ser. II apparatus, fitted with a fused silica DB-5 capillary column (30 m × 0.25 mm i.d., 0.33-μm film thickness), coupled to an Agilent Mass Selective Detector MSD 5973; ionization energy voltage: 70 eV; electron multiplier voltage energy: 2000 V. Mass spectra were scanned in the range 40–500 amu, with a scan time of 5 scans/s. The gas chromatographic conditions were as reported in the previous paragraph; transfer line temperature: 295 °C.

### 3.5. Identification of the Essential Oil Components

The identification of the essential oil constituents was based on the comparison of their Kovats retention indices (RIs), determined relative to the tR values of *n*-alkanes (C_10_–C_35_) on both capillary columns with those in literature [17,18,19,20] and their mass spectra with those of authentic compounds available in our laboratories or those listed in the NIST 02 and Wiley 275 mass spectral libraries [21]. For some compounds, the identification was confirmed by coinjection with an authentic sample (Table 1).

### 3.6. Determination of Minimum Inhibitory Concentration and Minimum Bactericidal Concentration 

The antibacterial activity was evaluated by determining the minimum inhibitory concentration (MIC) and the minimum bactericidal concentration (MBC) using the broth dilution method [22]. Ten bacteria strains, selected as representative of the class of Gram-positive and Gram-negative, were tested: *Staphylococcus aureus* (ATTC 25923), *Streptococcus faecalis* (ATTC 29212), *Bacillus cereus* (ATCC 1177), *B. subtilis* (ATCC 6633), *Escherichia coli* (ATCC 25922), *Pseudomonas aeruginosa* (ATCC 27853), *Staphylococcus epidermidis* (ATCC 12228), *Klebsiella pneumoniae* (ATCC 10031), *Salmonella typhi* Ty2 (ATCC 19430) and *Proteus vulgaris* (ATCC 13315). The strains were maintained on Tryptone Soya agar (Oxoid, Milan, Italy); for the antimicrobial tests, Tryptone Soya broth (Oxoid, Milan, Italy) was used. In order to facilitate the dispersion of the oil in the aqueous nutrient medium, it was diluted with Tween 20, at a ratio of 10%. Each strain was tested with sample that was serially diluted in broth to obtain concentrations ranging from 100 μg/mL down to 0.8 μg/mL. The sample was previously sterilized with a Millipore filter of 0.20 μm. The samples were stirred, inoculated with 50 μL of physiological solution containing 5 × 10^6^ microbial cells, and incubated for 24 h at 37 °C. The MIC value was determined as the lowest concentration of the sample that did not permit any visible growth of the tested microorganism after incubation. The control containing only Tween 20 was not toxic to the microorganisms. As positive controls, cultures containing only sterile physiological solution Tris buffer were used. MBC was determined by subculture of the tubes with inhibition in 5 mL of sterile nutrient broth. After incubation at 37 °C, the tubes were observed. When no growth was observed, the sample denoted a bactericidal action. The oil sample was tested in triplicate. Chloramphenicol was used as the standard antibacterial agent.

### 3.7. Determination of Total Phenolics

The total phenolic content was determined following the microscale protocol for Folin-Ciocalteu colorimetry, an alternative protocol for small sample volumes [23]. Each oil sample (20 μL, dissolved in ethanol, to obtain a final concentration of 50 mg/5 mL), a gallic acid calibration standard (50 mg/mL; 100 mg/mL; 250 mg/mL; 500 mg/mL) or blank (distilled water) was taken in a test cuvette. The absorbance was determined at room temperature at k = 765 nm using a Cary UV/Vis spectrophotometer (Varian Cary 50 MPR). The quantification was based on a standard curve generated with gallic acid; the results were expressed as mg gallic acid equivalents (GAE)/g of essential oil. A methanolic solution of gallic acid was tested in parallel as a reference compound.

### 3.8. Antioxidant Activity

The antiradical activity of the extracts under investigation was determined using the stable 1,1-diphenyl-2-picrylhydrazyl radical (DPPH), according to the method reported by Brand-Williams and coworkers [24] with some modifications to adapt the procedure using 96-well microplates [25]. In its radical form, DPPH has an absorption band at 517 nm, which disappears upon reduction by an antiradical compound. Briefly, an aliquot (7 μL) of the MeOH solution containing different amounts of the oils was added to 280 μL of DPPH solution (7.6 × 10^−5^ M), prepared daily, kept in the dark when not used. An equal volume (7 μL) of the vehicle alone was added to control tubes. Absorbances at 517 nm were measured on a Multiskan Spectrum Microplate Spectrophotometer (Thermo Fischer Scientific, Vantaa, Finland) 0, 10, 20, 30, 40, 50 and 60 min after starting the reaction. For preparation of the standard curve, different concentrations of DPPH methanol solutions (5–40 μg/mL) were used. Moreover, the solution of ascorbic acid was used for a calibration curve of DPPH reduction and as a chemical reference in comparison to the antioxidant capacities of the oils. Ascorbic acid was obtained from Fluka (Buchs, Switzerland). Ascorbic acid is an effective antioxidant [26]. Ascorbic acid was solved in methanol to have the following final concentrations (5 μg/mL, 2.5 μg/mL, 1.25 μg/mL, 0.625 μg/mL, 0.3125 μg/mL). The DPPH concentration (μg/mL) in the reaction medium was calculated from the following calibration curve, determined by linear regression (*r*^2^: 0.9974):

Absorbance (λ_517_) = 0.00186 + 0.0187 × [DPPH]

The IC_50_ value was defined as the concentration of sample that reduced the initial DPPH concentration by 50%, as compared to the negative control.

### 3.9. Statistical Analysis

Data from the determination of total phenolics were analyzed in GraphPad Prism 6.0 for correlation and significance (one-way ANOVA and Dunnett’s multiple comparison *post*-test). Data on antioxidant activity are expressed as the mean ± SD of five experiments.

## 4. Conclusions

The results reported here may help to shed light on the apparently complex chemotaxonomy of the genus *Thymus*. All five samples belong to the thymol chemotype, showing a homogeneity of prevalent monoterpenes in the oils. This finding seems to be related to the circum-Mediterranean distribution of this chemotype, which is the only one with the characteristic flavor and aroma of true thyme. Moreover, this study focused on the phenolic fraction and the effectiveness of *T. vulgaris* essential oils as an antimicrobial and antioxidant. Therefore, these oils could be used in many fields as natural preservatives of food and as nutraceuticals.
